# Ultrasonographic and Elastographic Markers of Muscle Changes Associated with Post-Stroke Spasticity: A Systematic Review

**DOI:** 10.3390/healthcare14203492

**Published:** 2026-10-17

**Authors:** Livia-Alexandra Ion, Delia Cinteză, Daniela Poenaru, Claudia Gabriela Potcovaru, Andreea Dumitrescu, Florina Ligia Popa

**Affiliations:** 1 Doctoral School, Lucian Blaga University of Sibiu, 550024 Sibiu, Romania; 2 National Institute of Rehabilitation, Physical Medicine and Balneoclimatology, 030079 Bucharest, Romania; daniela.poenaru@umfcd.ro (D.P.); gabriela.potcovaru@umfcd.ro (C.G.P.); andreea.petrut@drd.umfcd.ro (A.D.); 3 Physical Medicine and Rehabilitation, “Carol Davila” University of Medicine and Pharmacy, 050474 Bucharest, Romania; 4 Doctoral School, Carol Davila University of Medicine and Pharmacy, 020021 Bucharest, Romania; 5 Department of Physical Medicine and Rehabilitation, Faculty of Medicine, Lucian Blaga University of Sibiu, 550024 Sibiu, Romania; florina-ligia.popa@ulbsibiu.ro; 6 Sibiu County Emergency Clinical Hospital, 550245 Sibiu, Romania

**Keywords:** post-stroke spasticity, stroke rehabilitation, musculoskeletal ultrasound, B-mode ultrasonography, ultrasound elastography, shear wave elastography, muscle stiffness, muscle architecture, Modified Ashworth Scale, skeletal muscle

## Abstract

**Background/Objectives:** Clinical assessment of post-stroke spasticity may not fully distinguish neural contributions from structural and mechanical muscle changes. This systematic review examined associations between B-mode ultrasonographic and elastography-derived muscle parameters and the presence or severity of post-stroke spasticity. **Methods:** PubMed, Scopus, and Web of Science Core Collection were searched from inception to 18 July 2026. Eligible studies investigated ultrasound-derived skeletal muscle parameters in adults after stroke in relation to spasticity status or severity. Methodological quality was assessed using the Joanna Briggs Institute checklist, and certainty of evidence was evaluated using the GRADE approach. Findings were synthesized narratively because of substantial heterogeneity. **Results:** Eighteen studies were included, six contributing B-mode structural data and twelve elastography-derived mechanical data. B-mode findings were inconsistent across muscles and measurement conditions. Elastography more frequently identified increased stiffness-related parameters in paretic or spastic muscles, although associations with clinical spasticity severity were not consistently observed. The certainty of evidence was very low across the assessed outcome domains, reflecting methodological limitations, small sample sizes, and inconsistency in the reported associations. **Conclusions:** B-mode ultrasound and elastography provide complementary information on muscle changes after stroke, but neither can currently serve as a stand-alone measure of spasticity severity. Elastography may help characterize the mechanical component of post-stroke hypertonia alongside clinical examination. Larger, standardized longitudinal studies are needed to establish the clinical utility of these techniques.

## 1. Introduction

Post-stroke spasticity is a clinically important consequence of stroke and may contribute to pain, reduced range of motion, contracture, impaired mobility, upper-limb dysfunction, increased care burden, and lower quality of life [1]. In routine practice, spasticity is most often assessed using clinical scales such as the Modified Ashworth Scale and the Modified Tardieu Scale [2]. Although these scales are practical and widely used, they remain examiner-dependent and may not fully separate neural hyperexcitability from non-neural changes in muscle and soft tissue [3].

This distinction is clinically relevant because increased resistance to passive movement after stroke is not explained by reflex activity alone [4]. Over time, skeletal muscle may undergo structural and mechanical adaptations, including changes in muscle thickness, cross-sectional area, fascicle length, pennation angle, echogenicity, passive stiffness, and viscoelastic behaviour [5,6,7]. These peripheral changes may influence clinical examination findings and may also affect rehabilitation planning, treatment response, and the risk of developing fixed shortening or contracture [8].

Musculoskeletal ultrasonography offers a non-invasive and accessible way to assess these muscle changes. B-mode ultrasound can describe muscle morphology through parameters such as thickness, cross-sectional area, fascicle length, pennation angle, and echogenicity [9,10]. Ultrasound elastography, including shear wave and strain elastography, can provide information on mechanical properties such as shear wave velocity, Young’s modulus, shear modulus, stiffness, strain, or strain ratio [11]. Together, these techniques may complement clinical assessment by providing muscle-specific information that cannot be obtained from ordinal spasticity scales alone. Importantly, ultrasound-derived structural and mechanical parameters should not be regarded as direct measures of spasticity. B-mode ultrasonography characterizes muscle morphology, whereas elastography provides estimates of tissue mechanical properties under specific measurement conditions. Although these parameters may reflect muscle changes associated with post-stroke hypertonia, they do not directly quantify the neural, velocity-dependent component of spasticity. Their interpretation therefore requires consideration of clinical findings, muscle activation, joint position, and other factors influencing passive resistance to movement. Ultrasound should consequently be viewed as a complementary tool for characterizing muscle alterations rather than as a stand-alone method for diagnosing or grading spasticity.

However, the available studies are difficult to compare. They differ in post-stroke phase, assessed muscle, ultrasound modality, patient position, joint angle, measurement condition, comparator group, clinical spasticity scale, and statistical approach. As a result, it remains unclear which ultrasound-derived parameters are most consistently associated with the presence or severity of post-stroke spasticity [11].

Previous reviews have addressed different aspects of ultrasound-based muscle assessment, including the reliability and validity of elastography in neurological populations, its potential applications in post-stroke spasticity assessment and treatment monitoring, and the clinical validity of shear wave elastography [12,13,14]. However, these reviews have generally examined specific techniques or clinical applications rather than jointly considering B-mode structural parameters and elastography-derived mechanical properties in relation to post-stroke spasticity. The present review aims to address this gap by integrating evidence from both modalities across upper- and lower-limb muscles, while distinguishing ultrasound findings directly associated with clinical spasticity from muscle changes identified through paretic versus non-paretic or healthy-control comparisons. This approach allows a broader assessment of the available evidence and its limitations, particularly regarding the interpretation of ultrasound-derived parameters as potential adjuncts to clinical spasticity assessment.

Therefore, the aim of this systematic review was to identify, critically appraise, and synthesize evidence on the association between B-mode musculoskeletal ultrasonographic and ultrasound elastography-derived skeletal muscle parameters and the presence or severity of post-stroke spasticity in adults. Secondary aims were to describe differences in ultrasound-derived parameters according to comparator type, ultrasound modality, muscle group, measurement condition, clinical spasticity scale, and post-stroke phase.

## 2. Materials and Methods

### 2.1. Protocol and Reporting Guidelines

This systematic review was conducted according to a pre-specified protocol and reported in accordance with the Preferred Reporting Items for Systematic Reviews and Meta-Analyses (PRISMA) 2020 statement [15]. The protocol was registered in the International Prospective Register of Systematic Reviews, PROSPERO, under registration number CRD420261432760. The PROSPERO record was created on 24 June 2026 and last edited on 14 July 2026. The protocol defined the review question, eligibility criteria, search strategy, data extraction items, risk-of-bias assessment, and synthesis approach.

### 2.2. Eligibility Criteria

Eligibility criteria were structured using an adapted Population, Exposure, Comparator, and Outcome (PECO) framework [16]. The population of interest included adults aged 18 years or older with a confirmed diagnosis of ischaemic or haemorrhagic stroke, with or without documented spasticity, across any post-stroke phase. The exposure or index methods of interest were B-mode musculoskeletal ultrasonography and/or ultrasound elastography assessment of skeletal muscle. Eligible comparators included spastic versus non-spastic muscles, paretic versus non-paretic muscles, healthy controls, severity strata, longitudinal change, or correlations with clinical spasticity measures. Eligible outcomes included structural or mechanical ultrasound-derived skeletal muscle parameters reported in relation to post-stroke spasticity presence, severity, clinical comparison, or relevant comparator groups.

Studies were included if they met the following criteria: adult post-stroke participants; assessment of skeletal muscle using B-mode ultrasound and/or ultrasound elastography; reporting of ultrasound-derived structural or mechanical muscle parameters; and analysis involving spasticity status, spasticity severity, spastic versus non-spastic comparison, paretic versus non-paretic comparison, healthy-control comparison, severity strata, or longitudinal change. Comparisons based only on paretic versus non-paretic muscles or healthy controls were retained as post-stroke muscle alteration data and were not interpreted as direct evidence of spasticity association unless linked to spasticity status or severity. These studies were retained to provide context on structural and mechanical muscle alterations after stroke that may contribute to the non-neural component of increased resistance to passive movement. Although such comparisons cannot establish an association with spasticity in the absence of a direct clinical assessment, they may help characterize the peripheral muscle changes relevant to post-stroke hypertonia. Accordingly, findings based solely on paretic versus non-paretic muscles or healthy-control comparisons were considered supportive contextual evidence and were distinguished from studies directly examining spasticity status or severity in the synthesis and interpretation of results.

Eligible clinical spasticity measures included the Modified Ashworth Scale, Modified Tardieu Scale, Tardieu Scale, Composite Spasticity Scale, or comparable clinical assessments. Studies were excluded if they included children or adolescents, non-stroke causes of spasticity, unclear or insufficiently confirmed stroke diagnosis, no musculoskeletal ultrasound assessment, vascular ultrasound only, or imaging modalities other than ultrasound or elastography. Studies reporting only functional outcomes without ultrasound-derived skeletal muscle parameters, only reliability or reproducibility data without relevant spasticity association or comparator data, or ultrasound parameters unrelated to skeletal muscle were also excluded. Reviews, editorials, protocols without results, case reports, case series with fewer than five participants, conference abstracts without complete data, grey literature, non-English articles, and treatment-effect-only analyses without extractable baseline or observational association data were excluded.

### 2.3. Information Sources and Search Strategy

The database searches were conducted in PubMed, Scopus, and Web of Science Core Collection between 16 and 18 July 2026, with the final search completed on 18 July 2026. No publication date restriction was applied. Only studies published in English were included. Additional eligible studies were identified by screening the reference lists of included studies and relevant reviews, and by forward citation searching where appropriate [17].

The search strategy combined terms related to stroke, post-stroke spasticity, musculoskeletal ultrasonography, ultrasound elastography, and skeletal muscle. No study-design filter was applied in order to maximize sensitivity. The final searches were developed from the protocol search concepts and implemented as database-specific free-text strategies to maintain consistency across PubMed, Scopus, and Web of Science Core Collection.

To assess the sensitivity of the search strategy, an additional verification search was performed using broader terminology for ultrasound elastography, including technique-specific terms and acronyms such as shear wave elastography, shear wave velocity, acoustic radiation force impulse, shear modulus, and Young’s modulus. This supplementary search was conducted to assess whether alternative elastography terminology, used inconsistently across the literature, identified additional eligible studies not captured by the main search strategy or citation searching. No additional eligible studies were identified. The complete primary and verification search strategies, including platform, search date, full search string, and number of records retrieved, are provided in Supplementary Table S1.

### 2.4. Study Selection

Search results were exported into reference management software, and duplicates were removed before screening. Before formal screening, the eligibility criteria were piloted on a small sample of records to ensure consistent interpretation between reviewers. Study selection was performed in two stages: title and abstract screening followed by full-text assessment. Titles and abstracts were screened independently by two reviewers against the predefined eligibility criteria. Potentially eligible articles were then assessed in full text by the same two reviewers. Disagreements were resolved through discussion and, when necessary, by consultation with a third reviewer. Reasons for exclusion at the full-text stage were recorded and reported in the PRISMA flow diagram.

### 2.5. Data Extraction

Data were extracted using a standardized, predefined data extraction form. The form was piloted on a small sample of eligible or potentially eligible studies and refined before full extraction. Data extraction was performed independently by two reviewers. Disagreements were resolved through discussion or, when necessary, by consultation with a third reviewer.

Extracted data included study characteristics, population characteristics, post-stroke phase, ultrasound method, B-mode structural parameters, elastography-derived mechanical parameters, spasticity measures, comparators, effect estimates, reliability or reproducibility data, and methodological quality information. Post-stroke phase was extracted as reported by each study. When exact time since stroke was available, it was retained; otherwise, the authors’ reported phase category was used. Because definitions of acute, subacute, and chronic stroke varied across studies, these categories were interpreted descriptively rather than as strictly standardized time windows.

Ultrasound-related data included modality, device or vendor, probe type and frequency, examiner training, assessor blinding, assessed muscles, assessed side, participant position, joint angle, measurement condition, number of measurements, and averaging method. Extracted B-mode parameters included muscle thickness, cross-sectional area, echogenicity or echo intensity, fascicle length, and pennation angle. Elastography-derived parameters included shear wave velocity or speed, Young’s modulus, shear modulus, stiffness, strain, and strain ratio.

Effect estimates included correlation coefficients, mean differences, standardized mean differences, confidence intervals, *p*-values, and other reported measures of association where available.

### 2.6. Methodological Quality Assessment

Methodological quality was assessed using the Joanna Briggs Institute critical appraisal checklist for analytical cross-sectional studies [18]. Critical appraisal was performed independently by two reviewers, with disagreements resolved through discussion or, when necessary, by consultation with a third reviewer. Item-level judgements were recorded for each study, and an overall judgement of low, moderate, or high methodological concern was assigned to support interpretation of the evidence synthesis.

Because the JBI checklist does not prescribe an overall numerical score or categorical cut-off, these overall categories represented review-level qualitative judgements rather than formal JBI-derived scores. Studies were rated as low concern when most domains were judged as adequate and no major limitation was identified in exposure measurement, outcome measurement, or statistical analysis. Studies were rated as moderate concern when one or more domains were unclear, partially reported, or limited, particularly regarding confounding control, assessor blinding, or sample description. Studies would have been rated as high concern if major limitations affected eligibility criteria, exposure measurement, outcome measurement, or statistical analysis.

Although some included reports had prospective, longitudinal, feasibility, or diagnostic-accuracy parent designs, the present review extracted baseline or cross-sectional observational association data between ultrasound-derived parameters and clinical spasticity measures. The appraisal tool was therefore selected according to the analytical component contributing data to the present synthesis rather than solely according to the parent study design label. Nevertheless, the use of a single appraisal framework across heterogeneous parent designs may not capture all design-specific sources of bias and was considered when interpreting the methodological quality assessment [19].

### 2.7. Data Synthesis

A structured narrative synthesis was performed because of clinical and methodological heterogeneity across studies. Findings were grouped by ultrasound modality, parameter type, muscle group, measurement condition, comparator type, clinical spasticity scale, and post-stroke phase where possible. Results were summarized according to the direction and magnitude of reported associations, together with measures of precision where available.

Findings were interpreted according to the type of comparison reported. Results based on correlations with clinical spasticity scales or comparisons between spastic and non-spastic muscles were considered direct spasticity-related findings. Results based only on paretic versus non-paretic or healthy-control comparisons were interpreted as post-stroke muscle alterations and were not considered direct evidence of association with spasticity unless they were also linked to spasticity status or severity.

Meta-analysis was not performed because no sufficiently homogeneous group of studies reported the same ultrasound modality, muscle, measurement condition, clinical comparator or spasticity scale, and effect estimate in a format suitable for pooling. In particular, studies differed in assessed muscle, joint position, resting versus stretch condition, elastography metric, comparator type, and reported statistical measure [20].

A descriptive direction-of-effect summary was also prepared to show whether reported associations indicated higher values, lower values, or no clear association with spasticity-related measures. This summary was used to support the narrative synthesis and was not interpreted as evidence of effect magnitude. Statistical significance was not used as a substitute for effect size.

### 2.8. Certainty of Evidence Assessment

The certainty of evidence was assessed using the GRADE approach, adapted for heterogeneous observational association evidence [21]. Because all included evidence was observational and no quantitative meta-analysis was performed, certainty was assessed narratively by outcome domain rather than through a formal Summary of Findings table based on pooled effect estimates. The initial certainty was considered low. The domains considered were methodological limitations, inconsistency, indirectness, imprecision, and possible publication bias or selective reporting. Downgrading was applied when concerns within a domain were judged sufficiently serious to reduce confidence in the overall body of evidence. Concerns that were present but not considered serious enough to warrant downgrading were described qualitatively. Certainty ratings were interpreted as structured narrative judgements rather than estimates derived from pooled effect sizes.

## 3. Results

### 3.1. Study Selection

**Figure 1:**
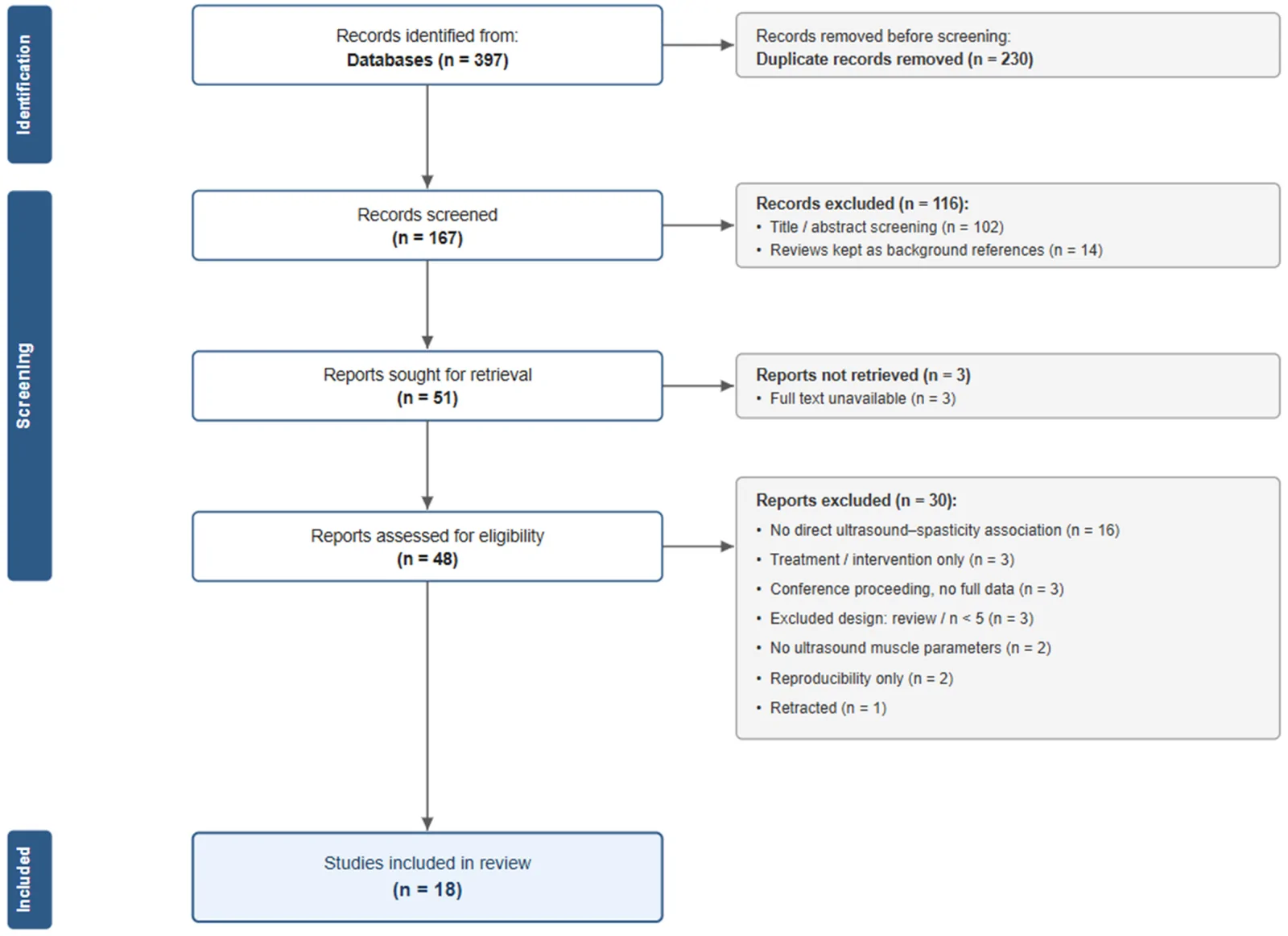
PRISMA 2020 flow diagram of the study selection process.

The database search identified 397 records. After removal of 230 duplicates, 167 records were screened by title and abstract. A total of 116 records were excluded at this stage. Fifty-one reports were sought for retrieval, of which three could not be obtained. Forty-eight full-text reports were assessed for eligibility, and 30 were excluded for predefined reasons. Finally, 18 studies were included in the systematic review. The study selection process is presented in Figure 1.

### 3.2. Characteristics of Included Studies

The 18 included studies were published between 2012 and 2025 and were conducted in Japan, Singapore, the United States, Hong Kong, Turkey, China, Spain, Italy, Taiwan, Brazil, and South Korea. Most studies had a cross-sectional design, while a smaller number were prospective, longitudinal, feasibility, or diagnostic accuracy studies. Sample sizes varied considerably, ranging from small feasibility studies with fewer than 10 stroke participants to larger observational cohorts of more than 60 stroke participants.

The studies included adults after stroke across different post-stroke phases, including acute–subacute, subacute, mixed subacute/chronic, and chronic stages. The most frequently assessed muscles were the biceps brachii and medial gastrocnemius, followed by the brachialis, flexor carpi radialis, brachioradialis, gastrocnemius lateralis, flexor carpi ulnaris, rectus femoris, and forearm flexors. Ultrasound methods included B-mode ultrasonography, shear wave elastography, strain elastography, and combined ultrasound protocols. Spasticity was most commonly assessed using the Modified Ashworth Scale, while some studies also used the Modified Tardieu Scale, Composite Spasticity Scale, or Spasticity Index. Two included reports from Gao et al. were considered companion reports from the same research group, with possible participant overlap. They were retained separately because they reported different ultrasound outcomes, but were interpreted cautiously in the synthesis. The main characteristics of the included studies are summarized in Table 1.

### 3.3. Methodological Quality

Five studies were judged as having low methodological concern, while thirteen studies were judged as having moderate methodological concern. No study was judged as having high methodological concern.

The most common methodological limitations were small sample size, limited adjustment for potential confounding factors, and incomplete reporting of assessor blinding. Overall, the included studies generally provided adequate descriptions of the study population, ultrasound or elastography protocol, clinical spasticity assessment, and statistical analysis.

The methodological quality assessment is summarized in Table 2.

BI checklist items: Q1, inclusion criteria clearly defined; Q2, study subjects and setting described; Q3, exposure measured validly and reliably; Q4, objective criteria used for measurement of the condition; Q5, confounding factors identified; Q6, strategies to deal with confounding factors stated; Q7, outcomes measured validly and reliably; Q8, appropriate statistical analysis used.

### 3.4. B-Mode Structural Findings

Six studies reported B-mode ultrasonographic structural parameters in relation to post-stroke spasticity [23,24,28,29,30,39]. Four reported at least one significant association with spasticity presence or clinical severity [23,28,30,39], whereas two found no significant association with the assessed clinical spasticity measures [24,29]. Among the studies reporting significant findings, the direction of the associations varied. Kong et al. reported differences in muscle thickness, echo intensity, and fascicle length according to spasticity presence [23]. Yang et al. found positive correlations between MAS and muscle thickness or pennation angle, but a negative correlation with fascicle length [28]. In contrast, Picelli et al. and Kim et al. reported reductions in selected architectural parameters with increasing spasticity severity [30,39]. Overall, B-mode findings were heterogeneous across muscles, parameters, and measurement conditions.

### 3.5. Elastography-Derived Mechanical Findings

Twelve studies reported elastography-derived mechanical parameters in relation to post-stroke spasticity [22,25,26,27,31,32,33,34,35,36,37,38]. Seven reported at least one significant association between an elastography-derived parameter and clinical spasticity scores or MAS-defined severity groups [25,26,31,32,33,37,38]. These findings included associations involving shear wave velocity, shear modulus, strain-based measures, and corrected slack angle, as well as differences in angle-dependent stiffness parameters across MAS groups. However, significant associations were not consistently observed for all parameters within individual studies. For example, Cao et al. reported a significant correlation between corrected slack angle and MAS, whereas the conventional shear modulus was not significantly associated with MAS [33]. Three studies found no significant association between the assessed elastography parameters and MAS or Tardieu scores [27,34,35], despite reporting muscle mechanical differences or associations with passive range of motion or biomechanical measures. One study reported strain-related differences between spastic and comparator muscles without providing a direct association with clinical severity [36]. One study found significant correlations between muscle–tendon junction displacement and MAS, but the elasticity index itself was not significantly associated with MAS [22]. A summary of the associations between ultrasound-derived parameters and clinical spasticity is presented in Table 3.

**Table 3:** Summary of associations between ultrasound-derived parameters and clinical spasticity.

Study	US Modality	Muscle(s)	Ultrasound Parameter(s)	Spasticity Scale	Main Association	Direction
**A. Structural B-mode findings**						
Kong 2022 [23]	B-mode	Brachialis	Muscle thickness, echo intensity, fascicle length	MAS	Spasticity presence at 1 month was associated with fascicle length (*p* = 0.002), muscle thickness (*p* = 0.017), and echo intensity (*p* = 0.023); at 6 months, fascicle length remained significant.	Mixed structural changes; higher thickness and fascicle length, lower echo intensity.
González-Buonomo 2023 [24]	B-mode	Biceps brachii; flexor carpi ulnaris; rectus femoris; medial gastrocnemius	Muscle thickness; echogenicity	MAS; Spasticity Index	Thickness versus Spasticity Index r = −0.13 to 0.19; echogenicity versus MAS r = 0.10 to 0.16; all *p* > 0.30.	No significant association.
Yang 2014 [28]	B-mode	Gastrocnemius/plantar flexors	Pennation angle, muscle thickness, fascicle length	MAS	Pennation angle rs = 0.55; muscle thickness rs = 0.72; fascicle length rs = −0.62; all *p* < 0.05.	Mixed: higher pennation angle and thickness, lower fascicle length with greater spasticity.
Pujol-Fuentes 2025 [29]	B-mode	Gastrocnemius medialis	Muscle thickness, pennation angle, echotexture	MAS	No significant influence of MAS on the assessed B-mode or texture parameters.	No significant association with severity.
Picelli 2014 [30]	B-mode	Gastrocnemius medialis and lateralis	Echo intensity, pennation angle, muscle thickness	MAS	Echo intensity rho = 0.32–0.34; pennation angle rho = −0.45 to −0.50; muscle thickness rho = −0.21 to −0.41; significant for echo, pennation angle, and part of thickness analyses.	Mixed: higher echo intensity, lower pennation angle and thickness with greater spasticity.
Kim 2012 [39]	B-mode	Medial gastrocnemius	Muscle thickness, pennation angle, fascicle length across MAS grades 1–3	MAS	Group comparisons by MAS grade were significant for muscle thickness, pennation angle, and fascicle length (*p* < 0.001).	Negative: thickness, pennation angle, and fascicle length decreased as MAS grade increased.
**B. Elastography-derived mechanical findings**						
Okazaki 2024 [22]	B-mode + strain elastography	Biceps brachii; medial gastrocnemius	Muscle-tendon junction displacement; elasticity index	MAS	Muscle-tendon junction displacement correlated with MAS: biceps brachii rho = 0.60; medial gastrocnemius rho = 0.71–0.72; *p* < 0.01. Elasticity index was not significant.	Positive for muscle-tendon junction displacement; no association for elasticity index.
Miller 2022 [25]	B-mode + SWE + Doppler	Biceps brachii; medial gastrocnemius	Shear modulus	Composite Spasticity Scale	Biceps brachii r = 0.35–0.46; medial gastrocnemius r = 0.26–0.37; *p* < =0.05.	Positive, weak-to-moderate.
Kesikburun 2015 [26]	B-mode + strain elastography	Medial and lateral gastrocnemius	Elasticity index and elasticity ratio	MAS	Medial gastrocnemius elasticity index r = 0.38 and elasticity ratio r = 0.40; *p* < 0.05.	Positive, weak.
Gao 2018a [27]	B-mode + SWE (VTIQ)	Biceps brachii	Shear wave velocity; grey-scale pixel value	MAS; Tardieu; passive ROM	Shear wave velocity versus MAS r2 approximately 0.29 (*p* = 0.17); versus Tardieu r2 = 0.06; versus passive ROM r approximately −0.88 (*p* < 0.0001).	No clear association with MAS/Tardieu; higher shear wave velocity associated with lower passive ROM.
Wu 2017 [31]	SWE (ARFI); B-mode	Biceps brachii	Shear wave velocity	MAS; MTS	Shear wave velocity correlated with MAS (rho = 0.66, *p* < 0.001) and MTS (rho = 0.54, *p* = 0.002).	Positive: higher shear wave velocity with greater clinical severity.
Zhang 2023 [32]	SWE	Biceps brachii	Young modulus-angle curve/curvature parameter alpha	MAS	Curvature parameter alpha increased across MAS groups (approximately 0.009 to 0.015; *p* < 0.001).	Positive: higher modulus and faster stiffening with greater MAS severity.
Cao 2022 [33]	2D-SWE	Medial gastrocnemius	Corrected slack angle; shear modulus	MAS	Corrected slack angle correlated strongly with MAS (R = −0.85, *p* < 0.001); plain modulus was not significant.	Negative: greater MAS associated with lower corrected slack angle; plain modulus not significant.
Lee 2015 [34]	B-mode + SWE (SSI)	Biceps brachii	Shear wave speed; echo intensity	MAS; Tardieu	Paretic muscle values were higher than non-paretic values, but no significant correlations with MAS or Tardieu were reported.	No clear association with MAS/Tardieu; paretic muscle values differed from non-paretic values.
Leng 2019 [35]	SWE	Flexor carpi radialis	Shear elastic modulus	MAS; NeuroFlexor	MAS-SWE association was not significant. SWE correlated with NeuroFlexor elasticity component (r = 0.57, *p* = 0.028) and viscosity component (r = 0.65, *p* = 0.009).	No clear association with MAS/Tardieu; paretic muscle values differed from non-paretic values.
Gao 2018b [36]	B-mode + strain elastography	Biceps brachii	Axial/longitudinal strain ratio; tissue velocity	MAS; Tardieu	Spastic versus healthy/non-spastic differences were reported (overall *p* < 0.0001; tissue velocity *p* = 0.005), but no MAS correlation coefficient was reported.	Lower strain and tissue velocity in spastic muscle, consistent with greater stiffness.
Wei 2022 [37]	B-mode + SWE	Biceps brachii	Shear wave speed at 45 and 90 degrees	MAS	Shear wave speed correlated with MAS at 45 degrees (r = 0.38) and 90 degrees (r = 0.40); both *p* < 0.05.	Positive: higher shear wave speed with greater MAS severity.
Galvao 2022 [38]	B-mode + SWE (SSI)	Brachioradialis; biceps brachii	Shear elastic modulus	MAS	Shear elastic modulus correlated with MAS for brachioradialis (R = 0.68) and biceps brachii (R = 0.68); *p* < 0.05.	Positive: higher shear modulus with greater MAS severity.

Abbreviations: ARFI, acoustic radiation force impulse; BB, biceps brachii; BR, brachioradialis; CSS, Composite Spasticity Scale; FCR, flexor carpi radialis; MAS, Modified Ashworth Scale; MG, medial gastrocnemius; MTS, Modified Tardieu Scale; NS, not significant; ROM, range of motion; SI, Spasticity Index; SSI, supersonic shear imaging; SWE, shear wave elastography; SWV/SWS, shear wave velocity/speed. Note: Reliability/agreement statistics were not treated as validity correlations. Coefficients are correlations with the clinical spasticity scale unless otherwise stated. Findings based on correlations with clinical spasticity scales or comparisons between spastic and non-spastic participants/muscles were considered direct spasticity-related evidence. Paretic versus non-paretic or healthy-control differences without an accompanying association with spasticity status or severity were interpreted as post-stroke muscle alterations rather than direct evidence of spasticity association.

### 3.6. Direction-of-Effect Summary

The descriptive direction-of-effect summary showed heterogeneous patterns across B-mode structural parameters. Six studies reported higher values of selected structural parameters with greater spasticity, others reported lower values, and others found no clear association with spasticity-related measures [23,24,28,29,30,39]. For muscle thickness, two studies reported higher values in association with spasticity [23,28], two reported lower values [30,39], and two found no clear association [24,29]. In contrast, among the twelve elastography studies, seven reported at least one significant association with clinical spasticity measures or MAS-defined severity groups [25,26,31,32,33,37,38]. Three studies [27,34,35] found no significant association between the assessed elastography parameters and clinical spasticity scores. The remaining two studies reported different patterns: one [36] identified strain-related differences between spastic and comparator muscles without reporting a direct correlation with clinical severity, whereas the other one found an association with MAS for muscle–tendon junction displacement, but not for the elasticity index [22].

The direction-of-effect counts presented in Table 4 summarize individual parameter categories and are not mutually exclusive across studies. Furthermore, differences between paretic and non-paretic muscles were distinguished from direct associations with clinical spasticity. Given the heterogeneity of the included studies, these findings should be interpreted descriptively rather than as evidence of superior performance of either ultrasound modality.

### 3.7. Heterogeneity of Ultrasound Protocols

Considerable clinical and methodological heterogeneity was observed across the included studies. Ultrasound acquisition protocols differed in terms of assessed muscle, ultrasound modality, participant position, joint angle, measurement condition, number of measurements, and averaging method. Clinical spasticity assessment also varied, although the Modified Ashworth Scale was the most frequently used scale. Studies further differed in comparator type and statistical approach.

Because of this heterogeneity, quantitative meta-analysis was not considered appropriate, and findings were synthesized narratively. Potential reporting bias due to missing results was considered qualitatively; formal assessment of small-study effects or publication bias using funnel plots or statistical tests was not performed because meta-analysis was not conducted and the included studies were clinically and methodologically heterogeneous. Planned subgroup and sensitivity analyses were not performed because of the small number of studies within each modality–muscle–measurement condition subgroup and the substantial heterogeneity of reported outcomes.

Details of ultrasound and elastography acquisition protocols, including device, probe, assessed muscle, side assessed, measurement condition, joint position, number of measurements, averaging method, and assessor blinding where reported, are provided in Supplementary Table S2.

### 3.8. Certainty of Evidence

The certainty of evidence was rated as very low across the assessed outcome domains. Although several studies reported associations between ultrasound-derived muscle parameters and post-stroke spasticity, confidence in these findings was limited by methodological concerns, small sample sizes, and variability in the reported results.

For B-mode structural parameters, the certainty of evidence was very low because of methodological limitations, imprecision, and inconsistent findings across muscles, post-stroke phases, and measurement conditions.

For elastography-derived mechanical parameters, evidence concerning the presence of spasticity was considered separately from evidence concerning clinical spasticity severity. Several studies reported stiffness-related differences in the expected direction, but confidence in these findings was limited by small samples, incomplete control of potential confounders, and differences in measurement protocols. The certainty of evidence was therefore rated as very low.

Associations between elastography-derived parameters and clinical spasticity severity were less consistent. Although several studies reported significant correlations with clinical scales, others found weak or non-significant associations. In addition, some elastography parameters were more closely related to passive range of motion or biomechanical measures than to MAS or Tardieu scores. These findings, together with methodological limitations and imprecision, supported a very low certainty rating. These assessments reflect confidence in the available evidence rather than the magnitude or direction of the observed associations. The outcome-specific judgments are summarized in Table 5.

## 4. Discussion

### 4.1. Main Findings

The available evidence suggests that musculoskeletal ultrasound can capture muscle changes related to post-stroke spasticity, but the pattern differs between structural and mechanical measurements. Elastography-derived parameters showed promising associations with spasticity-related muscle stiffness, although the findings were not uniform across studies.

Several studies reported higher shear wave velocity, shear modulus, Young’s modulus, or other stiffness-related parameters in spastic or paretic muscles, and some found significant positive correlations with clinical spasticity scales [22,25,26,27,31,32,33,34,35,36,37,38]. However, these associations were not uniform across studies, and some elastography measures appeared to relate more closely to passive range of motion or biomechanical stiffness than to MAS or Tardieu scores [27,35].

By contrast, B-mode structural findings were more variable. Some studies found associations between spasticity and muscle thickness, fascicle length, pennation angle, or echogenicity, whereas others did not [23,24,28,29,30,39]. Even when significant associations were reported, their direction was not consistent across muscles, parameters, and post-stroke phases. This suggests that B-mode ultrasound may be useful for describing muscle morphology after stroke, but structural parameters should not be interpreted as simple or direct markers of spasticity severity.

These findings should be interpreted in relation to previous syntheses. Earlier reviews have mainly focused on ultrasound elastography, shear wave elastography validity, or the reliability of B-mode muscle architecture measurements [7,11,14,40,41]. The present review adds a broader perspective by bringing together B-mode structural parameters and elastography-derived mechanical parameters, while considering both positive and non-significant findings in relation to spasticity presence or severity. This combined view highlights the complementary information provided by the two modalities. Although elastography-derived stiffness measures showed a more consistent direction of findings in the included studies, this observation should be interpreted cautiously given the unequal number of studies, substantial methodological heterogeneity, and limited direct comparisons between modalities.

The development and clinical expression of post-stroke spasticity may also be influenced by lesion topography. Previous neuroimaging studies have reported associations between spasticity and lesions involving the internal capsule, corona radiata, and other regions involved in motor control. These findings highlight the relevance of the anatomical structures affected, beyond vascular territory alone. Although the relationship between lesion location and ultrasound-derived muscle parameters remains unclear, differences in lesion topography may contribute to the clinical heterogeneity observed across studies [42,43].

### 4.2. Interpretation of B-Mode Structural Findings

The structural ultrasound findings indicate that post-stroke muscle changes are not uniform across studies. In some studies, greater spasticity was associated with increased muscle thickness or pennation angle and reduced fascicle length, while in others higher spasticity was associated with reduced muscle thickness, pennation angle, or fascicle length. For example, Yang et al. reported increased pennation angle and muscle thickness but decreased fascicle length with higher MAS scores [28], whereas Kim et al. reported decreases in muscle thickness, pennation angle, and fascicle length as MAS grade increased [39]. Picelli et al. found higher echo intensity but lower pennation angle and reduced thickness in relation to spasticity [30], while Kong et al. reported early changes in thickness, echo intensity, and fascicle length according to spasticity presence [23]. In Kong et al., lower echo intensity referred to the comparison between spastic and non-spastic patients, whereas echo intensity was higher on the hemiparetic side than on the intact side [23]. Together with greater thickness and longer fascicles, this pattern may indicate relative preservation of muscle mass at 1 month rather than poorer muscle quality.

These divergent patterns probably reflect differences in the studied muscles, timing after stroke, limb region, severity of impairment, and measurement condition [23,24,28,29,30,39]. Upper-limb and lower-limb muscles may adapt differently after stroke, and chronic spastic muscles may show a combination of altered muscle architecture, disuse-related atrophy, increased connective tissue, and changes in passive mechanical properties. Therefore, B-mode ultrasound appears useful for describing muscle morphology, but its interpretation as a direct marker of spasticity severity should be cautious.

### 4.3. Interpretation of Elastography-Derived Mechanical Findings

Elastography-derived measures provided information on muscle mechanical properties associated with post-stroke spasticity [22,25,26,27,31,32,33,34,35,36,37,38]. This was particularly evident in studies using shear wave elastography, where stiffness-related values were frequently higher in the paretic or spastic limb. Wu et al. found positive correlations between biceps brachii shear wave velocity and both MAS and Modified Tardieu Scale scores [31]. Wei et al. also reported positive correlations between shear wave speed and MAS at different elbow positions [37]. Galvão et al. found that shear elastic modulus of both brachioradialis and biceps brachii correlated with MAS [38]. Miller et al. reported weak-to-moderate positive correlations between shear modulus and Composite Spasticity Scale scores in the biceps brachii and medial gastrocnemius [25]. Kesikburun et al. found weak positive correlations between strain elastography parameters and MAS in the gastrocnemius [26]. Okazaki et al. also reported positive correlations between muscle–tendon junction displacement and MAS, whereas the elasticity index was not significantly associated with MAS [22].

Dynamic or angle-dependent elastography may be particularly informative. Zhang et al. assessed Young’s modulus during passive stretching and found that stiffness increased more rapidly with higher MAS severity [32]. Cao et al. reported a strong negative association between corrected slack angle and MAS, suggesting that altered passive muscle behaviour during stretch may provide clinically relevant information beyond a single resting stiffness value [33].

However, negative or non-significant findings are equally important. Gao et al. found no significant association between shear wave velocity and MAS or Tardieu scores, although shear wave velocity was strongly related to passive range of motion [27]. Lee et al. reported altered shear wave speed and echo intensity in paretic muscle, but no correlation with MAS or Tardieu scores [34]. Leng et al. found that shear elastic modulus correlated with biomechanical NeuroFlexor components but not with MAS [35]. These findings suggest that elastography may detect passive tissue stiffness, while MAS and Tardieu also reflect examiner-dependent clinical resistance, reflex activity, joint position, and velocity-dependent responses.

### 4.4. Ultrasound-Based Phenotyping of Peripheral Contributors to Post-Stroke Hypertonia

An important interpretation emerging from this synthesis is that musculoskeletal ultrasound may be better viewed not as a stand-alone biomarker of clinical spasticity severity, but as a tool for phenotyping the peripheral muscular component of post-stroke hypertonia. Clinical scales such as MAS and Tardieu quantify resistance to passive movement, but this resistance may reflect variable contributions from neural hyperexcitability, passive muscle stiffness, muscle shortening, altered architecture, and evolving contracture [5,44,45,46]. Therefore, imperfect correlations between ultrasound-derived parameters and clinical spasticity scores should not necessarily be interpreted as a failure of ultrasound-based assessment; rather, they may be biologically informative.

For example, high clinical spasticity scores with relatively low elastography-derived stiffness may suggest a stronger neural contribution [27,34,35], whereas high clinical scores combined with increased stiffness or a shortened slack angle may indicate a more substantial peripheral mechanical component [31,32,33,37,38]. Similarly, abnormal B-mode structural findings in patients with low or moderate clinical spasticity may reflect early muscle remodelling before severe hypertonia or fixed contracture becomes clinically evident [23,24,28,29,30,39]. This interpretation remains hypothetical, because the included studies were not designed to classify patients into mechanistic phenotypes. However, it provides a useful framework for understanding why B-mode and elastography findings may not align perfectly with MAS or Tardieu scores.

From this perspective, ultrasound and elastography may be most useful when interpreted alongside clinical examination, with the potential to support more individualized rehabilitation and focal treatment decisions. Future longitudinal studies should test whether combined clinical and ultrasound-based phenotyping can predict spasticity progression, contracture development, response to botulinum toxin, or rehabilitation outcomes.

### 4.5. Clinical Relevance

Clinically, the main value of musculoskeletal ultrasound and elastography may be their ability to complement, rather than replace, bedside spasticity assessment. MAS and MTS remain practical and widely used, but they do not fully distinguish reflex-mediated resistance from passive muscle stiffness, soft-tissue shortening, or evolving contracture [3,44].

Elastography may have potential to contribute when clinical examination alone cannot determine why resistance to passive movement is increased. Muscle-specific imaging may eventually complement clinical reasoning when planning focal interventions such as botulinum toxin injections, particularly when several muscles contribute to the same clinical posture. For example, studies assessing elbow flexors showed that biceps brachii, brachialis, brachioradialis, and forearm flexors may present different structural or mechanical patterns [23,25,31,34,35,37,38], suggesting that imaging may have potential to complement muscle selection and treatment planning.

B-mode ultrasound may be useful for monitoring longer-term structural muscle changes, such as muscle thinning, altered fascicle length, pennation angle, and increased echogenicity [23,24,28,29,30,39]. However, given the variability of B-mode findings across studies, these parameters should be interpreted together with clinical examination, elastography findings, joint range of motion, and functional status.

### 4.6. Methodological and Clinical Heterogeneity

A major finding of this review is the high methodological heterogeneity across studies. The included studies differed in ultrasound modality, muscle group, joint position, measurement condition, clinical spasticity scale, comparator, and statistical approach. The protocol anticipated this issue and planned a structured narrative synthesis grouped by modality, muscle group, measurement condition, comparator type, clinical scale, and post-stroke phase.

This heterogeneity limits direct comparison between studies and makes quantitative pooling difficult. For example, some studies measured muscles at rest, while others assessed passive stretch, full joint extension, different elbow or ankle angles, or dynamic conditions [27,31,32,33,34,37]. Some studies used the contralateral limb as the comparator, while others included healthy controls, non-spastic post-stroke patients, or severity-based MAS groups [23,27,29,31,32,33,34,36,37,39]. These methodological differences may partly explain why some studies found strong associations with spasticity scales, while others found weak or non-significant correlations. A further source of heterogeneity was that the included studies addressed two related but distinct questions. Some studies examined whether ultrasound-derived parameters differed according to the presence of spasticity, by comparing spastic muscles with non-spastic or healthy muscles [23,29,31,32,33,34,36,37], whereas others examined whether these parameters increased or decreased with clinical spasticity severity using graded MAS or Tardieu scores [25,26,28,30,35,38,39]. Therefore, presence-based and severity-based findings should not be interpreted interchangeably, and the direction-of-effect summary should be read as a descriptive overview rather than as a pooled estimate of association.

Another important issue is the use of clinical spasticity scales as reference standards. MAS was the most frequently used scale, but it is an ordinal clinical measure and may not fully capture the complexity of post-stroke hypertonia. In some studies, elastography correlated better with passive range of motion or biomechanical stiffness components than with MAS itself [27,35]. This suggests that future studies should combine clinical scales with biomechanical, functional, and imaging-based outcomes rather than relying on MAS alone.

Clinical heterogeneity should also be considered when interpreting these findings. Previous studies have reported sex-related differences in stroke risk factors, lesion distribution, stroke severity, and functional outcomes [47]. However, such differences do not necessarily imply sex-specific associations between ultrasound-derived muscle parameters and post-stroke spasticity. Although sex was included among the population characteristics specified for data extraction in our review protocol, sex-stratified analyses were not prespecified [48]. Consequently, the present review cannot establish whether these associations differ between women and men. Future studies should investigate this possibility and report sex-disaggregated findings where feasible.

### 4.7. Methodological Quality of Included Studies

Most included studies were judged as having moderate methodological concern [22,23,24,26,27,28,30,34,35,36,37,38,39], while five were judged as low concern [25,29,31,32,33]. No study was classified as high concern. The most frequent limitations were small sample size, limited control for potential confounding factors, and incomplete reporting of assessor blinding. Despite these limitations, most studies adequately described the study population, ultrasound or elastography protocol, clinical spasticity assessment, and statistical analysis.

These limitations should be considered when interpreting the findings. Small sample sizes reduce statistical power and may contribute to inconsistent results. Limited adjustment for confounders is particularly relevant because muscle stiffness and architecture may be influenced by age, sex, time since stroke, muscle disuse, rehabilitation exposure, range of motion limitation, prior botulinum toxin treatment, and severity of motor impairment [7,10,49,50]. Therefore, although the available evidence supports the relevance of ultrasound-derived muscle assessment, the certainty of conclusions remains limited by methodological constraints.

### 4.8. Limitations of This Review

This review has several limitations. First, the included studies were highly heterogeneous in terms of population, stroke phase, assessed muscles, ultrasound technique, measurement position, and clinical spasticity scale, which prevented quantitative meta-analysis. Second, most studies had small sample sizes, reducing precision, and cross-sectional or exploratory designs, limiting causal interpretation and the certainty of conclusion. Third, reporting of assessor blinding, reliability testing, and adjustment for potential confounders was incomplete in several studies. Fourth, the predominance of the Modified Ashworth Scale as the clinical comparator may have limited mechanistic interpretation, because resistance to passive movement reflects both neural and non-neural components [45,51]. In addition, the available data did not allow subgroup analyses according to stroke subtype or lesion topography, which may have contributed to the observed clinical heterogeneity.

Search-related limitations should also be acknowledged. Only full-text, peer-reviewed studies published in English were included, which may have introduced language and publication bias. In addition, the systematic search was limited to PubMed, Scopus, and Web of Science Core Collection, as Embase was not available, and grey-literature sources and trial registries were not searched systematically. Therefore, some relevant studies may have been missed. To mitigate this limitation, reference-list screening, forward citation searching, and a separate expanded elastography verification search across PubMed, Scopus, and Web of Science Core Collection were performed.

### 4.9. Future Directions

Future research should use standardized ultrasound and elastography protocols, including clearly defined muscle landmarks, joint angles, patient positioning, probe placement, number of measurements, and averaging methods [52,53]. Larger prospective studies are needed to determine how ultrasound-derived structural and mechanical parameters evolve from the acute or subacute stage to the chronic stage after stroke [50]. Studies should also include relevant confounders such as age, sex, time since stroke, stroke severity, range of motion, rehabilitation exposure, and previous antispastic treatment. Stroke subtype and lesion location should also be considered in future investigations of ultrasound-derived muscle changes. Pure motor stroke is frequently associated with lacunar infarction, which has generally been linked to a more favourable functional prognosis than non-lacunar stroke [54]. However, differences in functional outcomes do not necessarily imply corresponding differences in spasticity severity or muscle structural and mechanical properties. Future studies should investigate whether B-mode and elastography-derived parameters differ between patients with lacunar and non-lacunar stroke, while accounting for lesion location, stroke severity, and time since stroke.

Future studies should combine clinical scales with biomechanical and functional measures, rather than using MAS alone as the reference standard [44]. Dynamic or angle-dependent elastography during passive stretch may be particularly promising, as it may characterize changes in passive muscle stiffness that are not captured by a single resting measurement [32,33,37]. Longitudinal studies are also needed to determine whether early ultrasound or elastography findings can predict later severe spasticity, contracture, or need for focal treatment.

## 5. Conclusions

In conclusion, B-mode ultrasonography and ultrasound elastography provide complementary information about structural and mechanical muscle changes associated with post-stroke spasticity. B-mode findings were heterogeneous, suggesting that structural muscle changes vary according to muscle, stroke phase, and measurement condition. Elastography-derived mechanical parameters showed somewhat more consistent associations with spasticity-related stiffness, although they did not always correlate directly with MAS or Tardieu scores. These findings suggest that ultrasound, particularly elastography, may be a useful adjunct to clinical assessment, but further standardized and longitudinal research is needed before it can be integrated routinely into clinical decision-making. Rather than serving as stand-alone biomarkers of clinical spasticity severity, ultrasound-derived parameters may be most useful as part of a broader clinical phenotyping approach to characterize structural and mechanical contributors to post-stroke hypertonia and support individualized rehabilitation planning.

## Figures and Tables

**Table 1:** Characteristics of included studies.

Study	Country	Design	Participants (n)	Age, y	Stroke Phase	Muscle(s)	US Modality	Spasticity Scale(s)
Okazaki 2024 [22]	Japan	Cross-sectional	23	77.7 ± 12.6	Subacute	Biceps brachii; medial gastrocnemius	B-mode + strain elastography	MAS
Kong 2022 [23]	Singapore	Prospective longitudinal	50	57.2 ± 12.3	Acute–subacute (≤1 mo)	Brachialis	B-mode	MAS
González-Buonomo 2023 [24]	USA	Cross-sectional	28	57.4 ± 11.9	Chronic	Biceps brachii; FCU; rectus femoris; medial gastrocnemius	B-mode	MAS; Spasticity Index
Miller 2022 [25]	Hong Kong/China	Cross-sectional + reliability	65	60.9 ± 7.7	Chronic	Biceps brachii; medial gastrocnemius	B-mode + SWE + Doppler	Composite Spasticity Scale
Kesikburun 2015 [26]	Turkey	Cross-sectional feasibility	26	52.4 ± 15.6	Chronic	Medial and lateral gastrocnemius	B-mode + strain elastography	MAS
Gao 2018a [27]	USA	Prospective feasibility	7 stroke; 8 controls	Stroke: 59; controls: 49	Chronic	Biceps brachii	B-mode + SWE (VTIQ)	MAS; Tardieu
Yang 2014 [28]	China	Cross-sectional	41	54.6 ± 15.7	Subacute–early chronic	Gastrocnemius/plantar flexors	B-mode	MAS
Pujol-Fuentes 2025 [29]	Spain	Cross-sectional comparative	26 stroke; 26 controls	56.7 ± 11.8	Chronic	Gastrocnemius medialis	B-mode	MAS
Picelli 2014 [30]	Italy	Cross-sectional	43	61.3	Chronic	Gastrocnemius medialis and lateralis	B-mode	MAS
Wu 2017 [31]	Taiwan	Cross-sectional + reliability	31 stroke; 21 controls	60.3 ± 13.0	Acute–subacute (<6 mo)	Biceps brachii	SWE (ARFI); B-mode screening	MAS; MTS
Zhang 2023 [32]	China	Cross-sectional	54 stroke; 30 controls	59.7 ± 11.4	Subacute	Biceps brachii	SWE	MAS
Cao 2022 [33]	China	Cross-sectional diagnostic accuracy	20 stroke; 20 controls	52.3 ± 11.6	Subacute (<6 mo)	Medial gastrocnemius	2D-SWE	MAS
Lee 2015 [34]	USA	Cross-sectional	16	60.7 ± 8.0	Chronic	Biceps brachii	B-mode + SWE (SSI)	MAS; Tardieu
Leng 2019 [35]	China	Cross-sectional	15	54 ± 8	Mixed subacute/chronic	Flexor carpi radialis	SWE	MAS
Gao 2018b [36]	USA	Prospective feasibility	7 stroke; 8 controls	Stroke: 59; controls: 46	Chronic	Biceps brachii	Strain elastography + B-mode	MAS; Tardieu
Wei 2022 [37]	China	Cross-sectional	61 stroke; 24 controls	63.5 ± 12.7	Subacute (<6 mo)	Biceps brachii	B-mode + SWE	MAS
Galvão 2022 [38]	Brazil	Cross-sectional	11	55.6 ± 9.7	Chronic	Brachioradialis; biceps brachii	SWE (SSI); B-mode	MAS
Kim 2012 [39]	South Korea	Cross-sectional	30	~67.6	Chronic	Medial gastrocnemius	B-mode	MAS

Abbreviations: ARFI, acoustic radiation force impulse; CSS, Composite Spasticity Scale; FCU, flexor carpi ulnaris; MAS, Modified Ashworth Scale; MTS, Modified Tardieu Scale; NR, not reported; SSI, supersonic shear imaging; SWE, shear wave elastography; US, ultrasound; VTIQ, Virtual Touch Imaging Quantification. Gao 2018a and Gao 2018b were considered companion reports from the same research group, with possible participant overlap.

**Table 2:** Risk-of-bias assessment (JBI critical appraisal).

Study	Q1	Q2	Q3	Q4	Q5	Q6	Q7	Q8	Overall Judgement
Okazaki 2024 [22]	Y	Y	Y	Y	U	N	Y	Y	Moderate
Kong 2022 [23]	Y	Y	Y	Y	Y	N/U	Y	Y	Moderate
González-Buonomo 2023 [24]	Y	Y	Y	Y	Y	N/U	Y	Y	Moderate
Miller 2022 [25]	Y	Y	Y	Y	Y	Y/P	Y	Y	Low
Kesikburun 2015 [26]	Y	Y	Y	Y	U	N	Y	Y	Moderate
Gao 2018a [27]	Y	Y	Y	Y	Y	N/U	Y	Y	Moderate
Yang 2014 [28]	Y/U	Y	Y	Y	U	N/U	Y	Y/P	Moderate
Pujol-Fuentes 2025 [29]	Y	Y	Y	Y	Y	Y	Y	Y	Low
Picelli 2014 [30]	Y	Y	Y	Y	Y/P	N/U	Y	Y	Moderate
Wu 2017 [31]	Y	Y	Y	Y	Y/P	Y/P	Y	Y	Low
Zhang 2023 [32]	Y	Y	Y	Y	Y/P	Y/P	Y	Y	Low
Cao 2022 [33]	Y	Y	Y	Y	Y	Y/P	Y	Y	Low
Lee 2015 [34]	Y/U	Y	Y	Y	Y/P	Y/P	Y	Y/P	Moderate
Leng 2019 [35]	Y	Y	Y	Y	Y/P	N/U	Y	Y	Moderate
Gao 2018b [36]	Y	Y	Y/P	Y	Y/P	N/U	Y	Y/P	Moderate
Wei 2022 [37]	Y	Y	Y	Y	Y/P	N/U	Y	Y	Moderate
Galvão 2022 [38]	Y	Y	Y	Y	Y/P	N/U	Y	Y/P	Moderate
Kim 2012 [39]	Y	Y	Y	Y	Y/P	N/U	Y	Y/P	Moderate

Abbreviations: Y, yes; N, no; U, unclear; Y/P, yes/partial; Y/U, yes/unclear; N/U, no/unclear.

**Table 4:** Descriptive direction-of-effect summary of associations between ultrasound-derived parameters and post-stroke spasticity. Values are the number of studies reporting each direction; given the small number of studies per parameter, they are indicative.

Parameter	↑ With Spasticity	↓ With Spasticity	No Clear Association
**Structural outcomes (B-mode)**			
Muscle thickness	2	2	2
Pennation angle	1	2	1
Fascicle length	1	2	—
Echogenicity	1	1	2
**Mechanical outcomes (elastography)**			
Muscle stiffness (shear wave velocity, shear or Young’s modulus)	6	—	3

Note: Values represent parameter-level direction counts, not mutually exclusive study counts; individual studies could contribute to more than one parameter row. The stiffness row includes shear wave velocity/speed, shear modulus, and Young’s modulus. Strain-based parameters, muscle–tendon junction displacement, corrected slack angle, and angle-dependent parameters were summarized narratively because their direction is not directly comparable across measures. ↑ indicates higher parameter values associated with spasticity; ↓ indicates lower parameter values associated with spasticity.

**Table 5:** GRADE certainty of evidence assessment.

Evidence Domain	Risk of Bias	Inconsistency	Indirectness	Imprecision	Publication Bias	Overall Certainty
B-mode structural parameters and clinical spasticity	Serious	Serious	Not serious	Serious	Not formally assessed	Very low
Elastography-derived mechanical parameters and the presence of spasticity	Serious	Not serious	Not serious	Serious	Not formally assessed	Very low
Associations between elastography-derived parameters and clinical spasticity severity	Serious	Serious	Not serious	Serious	Not formally assessed	Very low

Notes: All included evidence was observational and was initially rated as low certainty. Certainty was assessed narratively at the outcome level because quantitative pooling was not appropriate. Differences between paretic and non-paretic muscles were not considered direct evidence of an association with spasticity unless linked to spasticity status or severity. Downgrading reflected methodological limitations, inconsistency where applicable, and imprecision. Publication bias could not be formally assessed.

## Data Availability

No new data were created or analyzed in this study. Data sharing is not applicable to this article.

## Supplementary Materials

The following supporting information can be downloaded at https://www.mdpi.com/article/10.3390/healthcare14203492/s1. Table S1: Complete database-specific search strategies; Table S2: Ultrasound and elastography acquisition protocols of included studies; Supplementary File S1: PRISMA 2020 checklist.
