# Supplementary material for: Ultrasonographic and Elastographic Markers of Muscle Changes Associated with Post-Stroke Spasticity: A Systematic Review
Source: Healthcare (Basel). 2026 Oct 17;14(20):3492. doi: 10.3390/healthcare14203492 (PMC13704180; doi:10.3390/healthcare14203492)
Supplement: Supplementary file 1 [file healthcare-14-03492-s001.zip › supplementary table.pdf]

Supplementary Table S1. Complete database-specific search strategies.

Primary database searches were used for the PRISMA record count. A separate verification/sensitivity search using expanded elastography terminology was performed on 25 August 2026 in the same three databases. No additional eligible studies were identified. Verification/sensitivity search records were not added to the PRISMA record count.

A. Primary systematic searches

Database	Platform	Search concept	Search date	Records retrieved
PubMed	PubMed/MEDLINE	B-mode / structural ultrasound	16 July 2026	62
**Full search string:** (stroke OR "post-stroke" OR "cerebrovascular accident") AND (spasticity OR hypertonia OR "muscle stiffness" OR "muscle tone") AND ("muscle architecture" OR "muscle thickness" OR "muscle echo intensity" OR echogenicity OR "fascicle length" OR "pennation angle" OR "cross-sectional area") AND (ultrasound OR ultrasonography OR sonography OR "B-mode")				
PubMed	PubMed/MEDLINE	Elastography / stiffness	16 July 2026	49
**Full search string:** (stroke OR "post-stroke" OR "cerebrovascular accident") AND (spasticity OR "muscle stiffness" OR hypertonia) AND (elastography OR "shear wave elastography" OR "shear wave velocity" OR "Young's modulus")				
Scopus	Scopus	B-mode / structural ultrasound	17 July 2026	70
**Full search string:** TITLE-ABS-KEY(stroke OR "post-stroke" OR "cerebrovascular accident") AND TITLE-ABS-KEY(spasticity OR hypertonia OR "muscle stiffness" OR "muscle tone") AND TITLE-ABS-KEY(ultrasound OR ultrasonography OR sonography OR "B-mode") AND TITLE-ABS-KEY("muscle architecture" OR "muscle thickness" OR "muscle echo intensity" OR echogenicity OR "fascicle length" OR "pennation angle" OR "cross-sectional area")				
Scopus	Scopus	Elastography / stiffness	17 July 2026	75
**Full search string:** TITLE-ABS-KEY(stroke OR "post-stroke" OR "cerebrovascular accident") AND TITLE-ABS-KEY(spasticity OR "muscle stiffness" OR hypertonia) AND TITLE-ABS-KEY(elastography OR "shear wave elastography" OR "shear wave velocity" OR "Young's modulus")				
Web of Science Core Collection	Web of Science	B-mode / structural ultrasound	18 July 2026	72
**Full search string:** TS=(stroke OR "post-stroke" OR "cerebrovascular accident") AND TS=(spasticity OR hypertonia OR "muscle stiffness" OR "muscle tone") AND TS=(ultrasound OR ultrasonography OR sonography OR "B-mode") AND TS=("muscle architecture" OR "muscle thickness" OR "muscle echo intensity" OR echogenicity OR "fascicle length" OR "pennation angle" OR "cross-sectional area")				
Web of Science Core Collection	Web of Science	Elastography / stiffness	18 July 2026	69
**Full search string:** TS=(stroke OR "post-stroke" OR "cerebrovascular accident") AND TS=(spasticity OR "muscle stiffness" OR hypertonia) AND TS=(elastography OR "shear wave elastography" OR "shear wave velocity" OR "Young's modulus")				

Total records before deduplication (primary searches only): 397

B. Verification/sensitivity searches: expanded elastography terminology

Database	Platform	Search concept	Search date	Records retrieved
PubMed	PubMed/MEDLINE	Expanded elastography verification / sensitivity search	25 August 2026	80
**Full search string:** (stroke[tiab] OR "post-stroke"[tiab] OR "cerebrovascular accident"[tiab] OR "cerebrovascular accidents"[tiab]) AND (elastograph*[tiab] OR sonoelastograph*[tiab] OR "shear wave"[tiab] OR SWE[tiab] OR SWV[tiab] OR ARFI[tiab] OR "acoustic radiation force impulse"[tiab] OR "shear modulus"[tiab] OR "Young's modulus"[tiab]) AND (muscle*[tiab] OR spastic*[tiab] OR hypertoni*[tiab])				
Scopus	Scopus	Expanded elastography verification / sensitivity search	25 August 2026	218
**Full search string:** TITLE-ABS-KEY(stroke OR "post-stroke" OR "cerebrovascular accident*") AND TITLE-ABS-KEY(elastograph* OR sonoelastograph* OR "shear wave" OR SWE OR SWV OR ARFI OR "acoustic radiation force impulse" OR "shear modulus" OR "Young's modulus") AND TITLE-ABS-KEY(muscle* OR spastic* OR hypertoni*)				
Web of Science Core Collection	Web of Science	Expanded elastography verification / sensitivity search	25 August 2026	114

Database	Platform	Search concept	Search date	Records retrieved
**Full search string:** TS=(stroke OR "post-stroke" OR "cerebrovascular accident*") AND TS=(elastograph* OR sonoelastograph* OR "shear wave" OR SWE OR SWV OR ARFI OR "acoustic radiation force impulse" OR "shear modulus" OR "Young's modulus") AND TS=(muscle* OR spastic* OR hypertoni*)				

Abbreviations: ARFI, acoustic radiation force impulse; SWE, shear wave elastography; SWV, shear wave velocity; TS, Topic Search; WoS, Web of Science.

Note: Verification/sensitivity searches were performed separately from the primary systematic search and were therefore not included in the PRISMA record count. These searches did not identify any additional eligible studies.

Supplementary Table S2: Ultrasound and elastography acquisition protocols of included studies.

Study	US modality	Device/vendor	Probe/frequency	Muscle(s) assessed	Side(s) assessed	Measurement condition / position	Elastography joint position / angle	No. measurements / averaging	Assessor blinding
Okazaki 2024	B-mode + sonoelastography / strain elastography	Aplio500, Toshiba	10 MHz linear probe	Biceps brachii; medial head of gastrocnemius	Paretic and non-paretic	Rest for thickness/echo intensity/elasticity index; passive joint movement for MTJ displacement	BB measured at 90° elbow flexion; MG measured at 30° ankle plantarflexion; MTJ displacement during 90°→45° elbow flexion and 30°→0° ankle plantarflexion	Once for each participant; repeated twice in 10 participants for intra-rater reliability	NR
Kong 2022	B-mode	Terason t3200	15–4 MHz linear array probe	Brachialis	Paretic and non-paretic	Rest/static measurement; supine position, elbow fully extended	NA	Three time points: baseline, 1 month, 6 months	N / not blinded to previous findings
González-Buonomo 2023	B-mode	NR	NR	Biceps brachii; flexor carpi ulnaris; rectus femoris; medial gastrocnemius	Paretic and non-paretic	Rest/static ultrasound assessment	NA	NR	NR
Miller 2022	B-mode + shear wave elastography + color Doppler	AixPlorer, Supersonic Imagine	4–15 MHz linear array transducer	Biceps brachii; medial gastrocnemius	Paretic and non-paretic	Passive/rest; participants relaxed, sEMG used to confirm passive muscle status	BB: elbow 60° flexion, shoulder 45° abduction; MG: ankle neutral 0° plantar-dorsiflexion	Average of 3 measures	ultrasound operator blinded to physical assessment results
Kesikburun 2015	B-mode + sonoelastography / strain elastography	GE LOGIQ S7, GE Healthcare	5–12 MHz linear transducer	Medial gastrocnemius; lateral gastrocnemius	Paretic and non-paretic	Rest; prone position, feet hanging off table, ankle in standardized resting position	Prone, feet hanging off table; ankle in standardized resting position	At least 3 compression–relaxation cycles for sonoelastography	Y; sonographer blinded to MAS scores
Gao 2018a	B-mode gray-scale + shear wave elastography / ARFI-VTIQ	Acuson S3000 HELX, Siemens	9L4 linear array transducer; gray-scale frequency 7 MHz	Biceps brachii muscle	Healthy BBM; post-stroke non-spastic BBM; post-stroke spastic BBM	Supine, arm relaxed, forearm supinated; elbow at 90° flexion and maximal achievable passive extension	90° elbow flexion and maximal achievable elbow extension	20 SWV measurements per BBM at each elbow angle; gray-scale pixel value measured twice	Y; scanning investigator blinded to MAS/TS, OT blinded to SWE results
Yang 2014	B-mode	Philips IU-22	L12-5 linear probe; exact MHz not clearly stated	Gastrocnemius / plantar flexor muscle region	Spastic/affected side and contralateral normal side; plus post-stroke normal tone control group	Prone, bilateral legs exposed, feet down at bedside, ankle neutral; gastrocnemius/plantar flexor region assessed	NA	NR	NR
Pujol-Fuentes 2025	B-mode ultrasound	Butterfly iQ+	NR	Gastrocnemius medialis	Stroke: affected and unaffected limbs; controls: dominant and non-dominant limbs	Seated, knees at 90° flexion	NA	Three transverse and three longitudinal images per participant	NR for assessor; image/textural analysis used standardized software workflow
Picelli 2014	B-mode	NR; same ultrasound machine model used at both centers	10 MHz linear transducer	Gastrocnemius medialis; gastrocnemius lateralis	Gastrocnemius medialis; gastrocnemius lateralis	Rest/static; prone position, legs outstretched	NA	NR	Y; ultrasound examiners blinded to EMG and clinical evaluations
Wu 2017	SWE / ARFI shear-wave velocity; B-mode used before SWV to exclude tear, hematoma, fibrotic changes	Acuson S2000, Siemens	7–9 MHz linear transducer, 9L4 Siemens	Biceps brachii	Stroke: paretic and non-paretic sides; controls: dominant and non-dominant sides	Rest/static; supine, arm relaxed, forearm supinated; elbow at 90° flexion and 0° extension; sEMG used to avoid active contraction	Elbow at 90° flexion and 0° extension; longitudinal axis most relevant	Five SWV measurements per side and elbow position, averaged	Partial: clinical assessor blinded to SWV; ultrasound examiner could not be truly blinded to paretic/non-paretic side
Zhang 2023	SWE	Aixplorer, Acoustics France	4–15 MHz linear array probe	Biceps brachii	Bilateral upper limbs; healthy controls, unaffected side of stroke patients, and affected side grouped by tone/MAS	Passive elbow extension using CPM device; seated position, shoulder flexed 90°	Passive elbow extension from approx. 80°/100° flexion to 180° extension; measurements across O/A–F elbow-angle positions	Two formal measurements per upper limb; Young's modulus recorded during passive elbow extension	NR
Cao 2022	2D-SWE	Aixplorer, Supersonic Imagine	4–15 MHz linear transducer, SuperLinear 15–4	Medial gastrocnemius	Stroke: spastic and unaffected sides; controls: matched side	Passive ankle movement; supine, knee flexed 30°, ankle moved by CPM from 40° plantarflexion to 21.5° dorsiflexion	Knee flexed 30°; ankle moved from 40° plantarflexion to 21.5° dorsiflexion; analysis at PF 25°, PF 5°, DF 15° and slack angle	Three SWE trials per session	Y for MAS assessors; MAS assessed by two blinded physical therapists; SWE examiners blinded to MAS during reliability testing
Lee 2015	Both: B-mode + shear wave ultrasound elastography / SSI	Aixplorer, SuperSonic Imagine	4–15 MHz linear transducer, SuperLinear 15–4	Biceps brachii	Paretic and non-paretic sides	Rest/static; seated, shoulder abducted 45°, elbow at 90°, forearm/wrist neutral; EMG monitored to avoid active contraction	Elbow positioned at 90° flexion	Five images per side	NR
Leng 2019	SWE	Aixplorer, Supersonic Imagine	4–15 MHz linear probe, SuperLinear 15-4	Flexor carpi radialis	Paretic and non-paretic sides	Passive stretch/rest condition; supine, upper limb relaxed; wrist angles 0°, 10°, 20°, 30°, 40°, 50°; EMG monitored to avoid active contraction	Wrist from 0° palmar flexion/rest to 50° dorsiflexion/passive stretch	Three trials of five images at each wrist angle	Partial; MAS and Fugl-Meyer assessed by physiotherapist blinded to experimental results
Gao 2018b	Strain elastography / ultrasound strain imaging; B-mode cine loops with 2D speckle tracking	Acuson S3000 HELX, Siemens	9L4 linear array transducer; scanning frequency 7 MHz	Biceps brachii	Healthy biceps; post-stroke non-spastic biceps; post-stroke spastic biceps	Passive/rest; supine, arm relaxed, forearm supinated; external compression with 1.0 kg sandbag; passive elbow extension/flexion	Elbow at 90° flexion and maximal extension; passive extension 90°→0°/maximal extension and flexion 0°→90°	Real-time US data recorded twice per muscle; interval 2 minutes	Y; ultrasound investigator blinded to MAS/Tardieu, occupational therapist blinded to strain imaging results
Wei 2022	Both: B-mode + SWE	Mindray Bio-Medical Electronics, Shenzhen, China	L11-3U linear transducer, 3–11 MHz	Biceps brachii muscle	Stroke: paretic and non-paretic sides; controls: dominant side	Rest/static; supine, elbow at 0° flexion; arm positioned at 45° and 90° relative to body	Arm lifted at 45° and 90° relative to body; elbow at 0° flexion; BBM stretched more at 90°	Three measurements/images per side, averaged	NR
Galvão 2022	SWE / Supersonic Shear Imaging; B-mode used for positioning	Aixplorer V.9, Supersonic Imaging	SuperLinear SL10-2 transducer, 2–10 MHz	Brachioradialis and biceps brachialis	Spastic/paretic side and contralateral non-spastic side	Rest/static; supine, shoulder abducted 10°, elbow at maximal extension/full extension where possible, forearm/wrist/fingers neutral	Elbow at full/maximal extension; one participant could only reach 60° elbow flexion	Two images per muscle; mean values used	NR
Kim 2012	B-mode	GE LOGIQ 3 EXPERT Color Doppler Ultrasound System	12 MHz linear transducer	Medial gastrocnemius	Paretic side	Rest and active/MVIC; sitting with legs outstretched; MVIC plantarflexion against dynamometer	NA	NR for ultrasound; MVIC measured 3 times and averaged	NR

Abbreviations: US, ultrasound; SWE, shear wave elastography; ARFI, acoustic radiation force impulse; SSI, supersonic shear imaging; BB, biceps brachii; MG, medial gastrocnemius; NR, not reported; NA, not applicable
